# Eosinophilic vacuolated tumor of the kidney misdiagnosed as upper tract urothelial carcinoma: a case report and literature review

**DOI:** 10.3389/fonc.2026.1739973

**Published:** 2026-04-20

**Authors:** Lihong Gu, Jingjing Huang, Yuqi Li, Zhang Tian, Yun Zhang, Yunjie Li, Lei Chen, Linfei Shu

**Affiliations:** 1 Department of First Urology, Zhuzhou Central Hospital, Zhuzhou, Hunan, China; 2 School of Medicine, Jishou University, Jishou, Hunan, China; 3 Department of Pathology, Zhuzhou Central Hospital, Zhuzhou, Hunan, China; 4 Department of Radiology, Zhuzhou Central Hospital, Zhuzhou, Hunan, China

**Keywords:** case report, eosinophilic vacuolated tumor, misdiagnosis, renal tumor, upper tract urothelial carcinoma

## Abstract

**Background:**

Eosinophilic vacuolated tumor (EVT) is a rare renal tumor subtype recognized in the 2022 WHO Classification of Urinary and Male Genital System Tumors under the category of other oncocytic renal tumors, though it has not yet been formally established as an independent diagnostic entity. Due to the lack of typical clinical and imaging features, preoperative diagnosis remains challenging. This article reports a case of EVT misdiagnosed as upper tract urothelial carcinoma (UTUC), aiming to improve clinical awareness of the atypical presentations of this tumor.

**Case summary:**

A 71-year-old male patient presented with painless gross hematuria for one week. He had a history of long-term smoking and renal calculi. CT revealed a soft tissue mass in the right renal pelvis region with marked enhancement during the corticomedullary phase and filling defect of the renal pelvis during the excretory phase. PET-CT showed a hypermetabolic nodule in the renal hilum region. Urine cytology revealed clusters of cells with nuclear atypia. Based on these findings, a preoperative diagnosis of UTUC was made, and laparoscopic right radical nephroureterectomy with bladder cuff excision was performed. Based on histological morphology and immunophenotype, the pathological diagnosis was eosinophilic vacuolated tumor (EVT). At 9-month follow-up, renal function remained unaffected, with no evidence of tumor recurrence or metastasis.

**Conclusion:**

EVT involving the renal sinus may present as a renal pelvis mass, which can be clinically misdiagnosed as UTUC. For renal pelvis tumors with atypical imaging features, preoperative biopsy should be actively pursued to establish a definitive pathological diagnosis and avoid overextension of surgical scope.

## 1 Introduction

Eosinophilic vacuolated tumor (EVT) is a rare renal tubular epithelial neoplasm. Due to its unique morphological and molecular genetic features, it was initially described as “high-grade oncocytic tumor” (1) or “sporadic renal cell carcinoma with eosinophilic and vacuolated cytoplasm” (2). In 2021, the Genitourinary Pathology Society (GUPS) unified its nomenclature as eosinophilic vacuolated tumor (3). Although EVT remains an emerging entity that has not yet been formally incorporated into the WHO classification system as a distinct diagnostic category, the 2022 WHO Classification of Urinary and Male Genital System Tumors (5th edition) acknowledges it under the category of other oncocytic renal tumors (4), reflecting ongoing efforts to better characterize this rare neoplasm.

Previous literature reports indicate that EVT mostly originates from the renal parenchyma and anatomically often presents as a mass in the upper or lower pole of the kidney (5, 6), with only a few cases involving the renal sinus (5, 7, 8). Upper tract urothelial carcinoma (UTUC) is a malignant tumor originating from the urothelium of the renal pelvis or ureter. While UTUC can range from low-grade, non-invasive lesions amenable to surgical cure to high-grade invasive tumors with poor prognosis, it differs fundamentally from EVT in pathological origin, biological behavior, and treatment approach. Although both are primary renal tumors, accurate differentiation is crucial for appropriate clinical management.

This article reports a rare case of EVT in which the tumor originated from the renal parenchyma and exhibited inward expansile growth with an endophytic pattern, involving the renal sinus fat and compressing the renal pelvis. On imaging studies, the mass demonstrated well-defined borders and was confined to the kidney, with a filling defect observed in the right renal pelvis on excretory phase imaging. These imaging features closely resembled UTUC, leading to preoperative misdiagnosis. Combined with relevant literature, we analyze and discuss the imaging features, pathological characteristics, and diagnostic challenges of this case, aiming to provide reference for differentiating EVT from UTUC in clinical practice.

## 2 Case report

### 2.1 Case presentation

**Figure 1:**
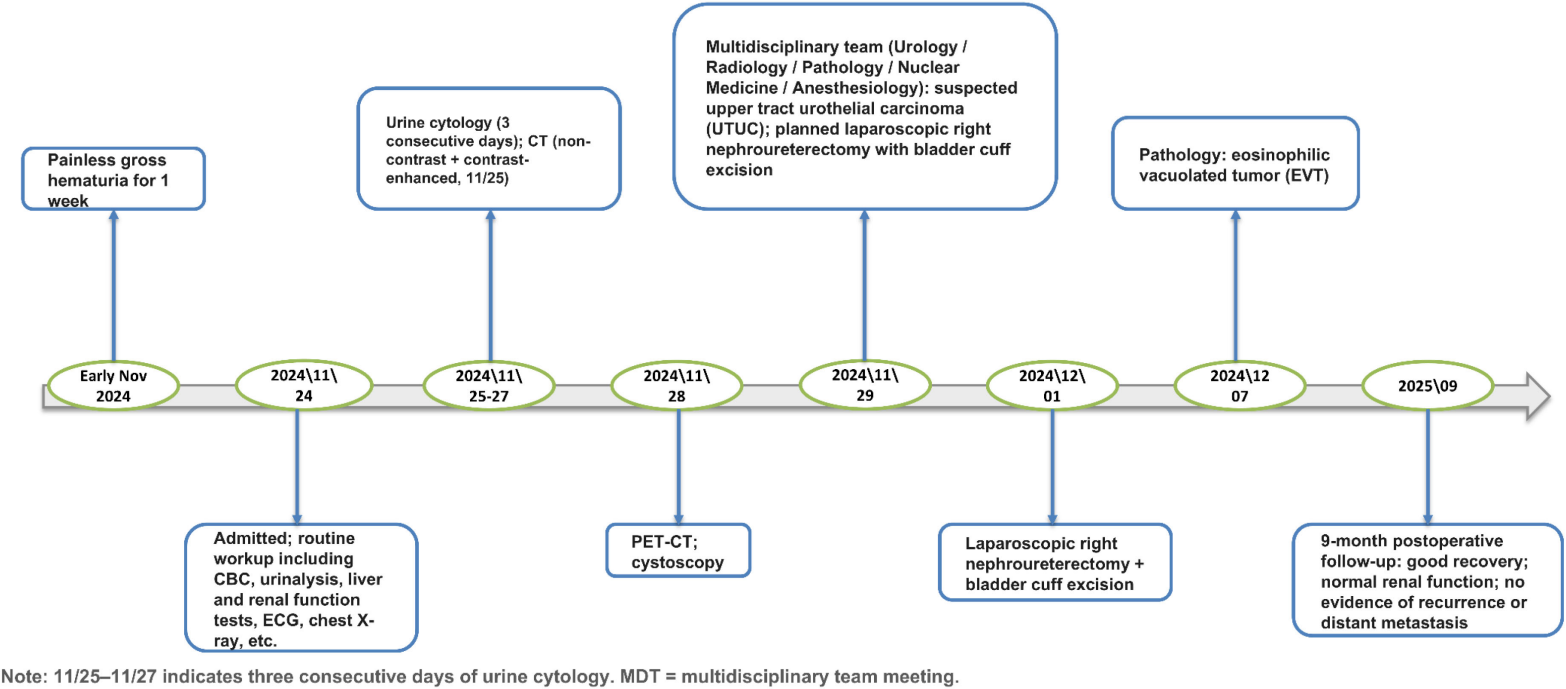
Timeline of diagnosis, treatment, and follow-up for a patient with eosinophilic vacuolated tumor of the kidney. Key events are presented chronologically from symptom onset to postoperative follow-up.

A 71-year-old male patient was admitted on November, 2024, with a chief complaint of “painless gross hematuria for one week.” One week prior to admission, the patient developed gross hematuria without obvious precipitating factors, presenting with dark red urine and blood clots, without urinary frequency, urgency, dysuria, or other irritative bladder symptoms, and without lumbago or fever. Past medical history included hypertension for 10 years, coronary heart disease for 5 years, and hemorrhagic stroke 1 year ago, all well-controlled with regular medication; no history of tuberous sclerosis complex. He had a smoking history of over 30 years, approximately 4–5 cigarettes per day. Urological physical examination revealed positive right renal area percussion tenderness (**Figure 1**). 

### 2.2 Laboratory examination

**Figure 2:**
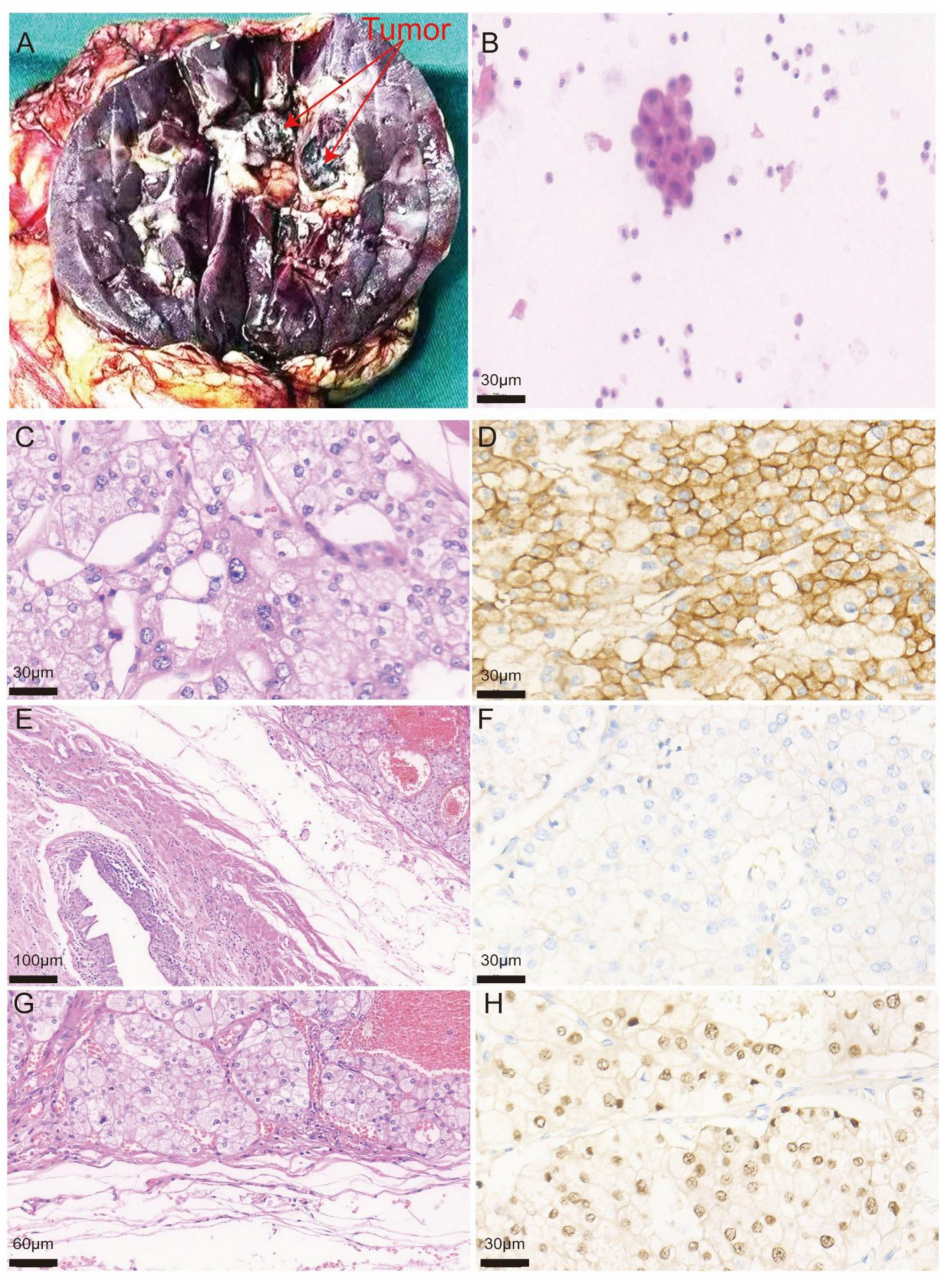
Gross, cytological, microscopic, and immunohistochemical findings of eosinophilic vacuolated tumor. **(A)** Gross specimen of the resected kidney showing a tan-brown tumor mass surrounded by yellow adipose tissue. The mass measured 2.6 cm × 2.0 cm × 1.7 cm, soft in consistency, with ill-defined margins, originating from the renal parenchyma and involving the renal sinus fat. **(B)** Urine cytology (×40) demonstrating clusters of atypical cells with nuclear pleomorphism, raising suspicion for malignant cells. **(C)** High-power microscopic view (×40, hematoxylin and eosin staining) showing tumor cells with abundant eosinophilic granular cytoplasm, characteristic intracytoplasmic vacuoles, round to oval nuclei, and prominent nucleoli. **(D)** CD117 immunostaining (×40) showing diffuse cytoplasmic and membranous positivity in tumor cells. **(E)** Low-power microscopic view (×10, hematoxylin and eosin staining) demonstrating tumor extension into renal sinus adipose tissue with compression but no invasion of the renal pelvis. **(F)** CK7 immunostaining (×40) showing negative staining in tumor cells. **(G)** Medium-power microscopic view (×20, hematoxylin and eosin staining) showing tumor infiltration into renal sinus adipose tissue. **(H)** PAX8 immunostaining (×40) showing diffuse nuclear positivity in tumor cells.

Urinalysis showed red blood cells 4+, white blood cells 1+; urine sediment microscopy revealed 460–500 red blood cells/HPF, 0–5 white blood cells/HPF; urine culture was negative. Urine cytology (**Figure 2B**) revealed several clusters of cells with nuclear atypia, not excluding tumor cells. Complete blood count, liver and renal function tests, electrolytes, coagulation profile, and prostate-specific antigen (PSA) were all within normal ranges.

### 2.3 Imaging examination

**Figure 3:**
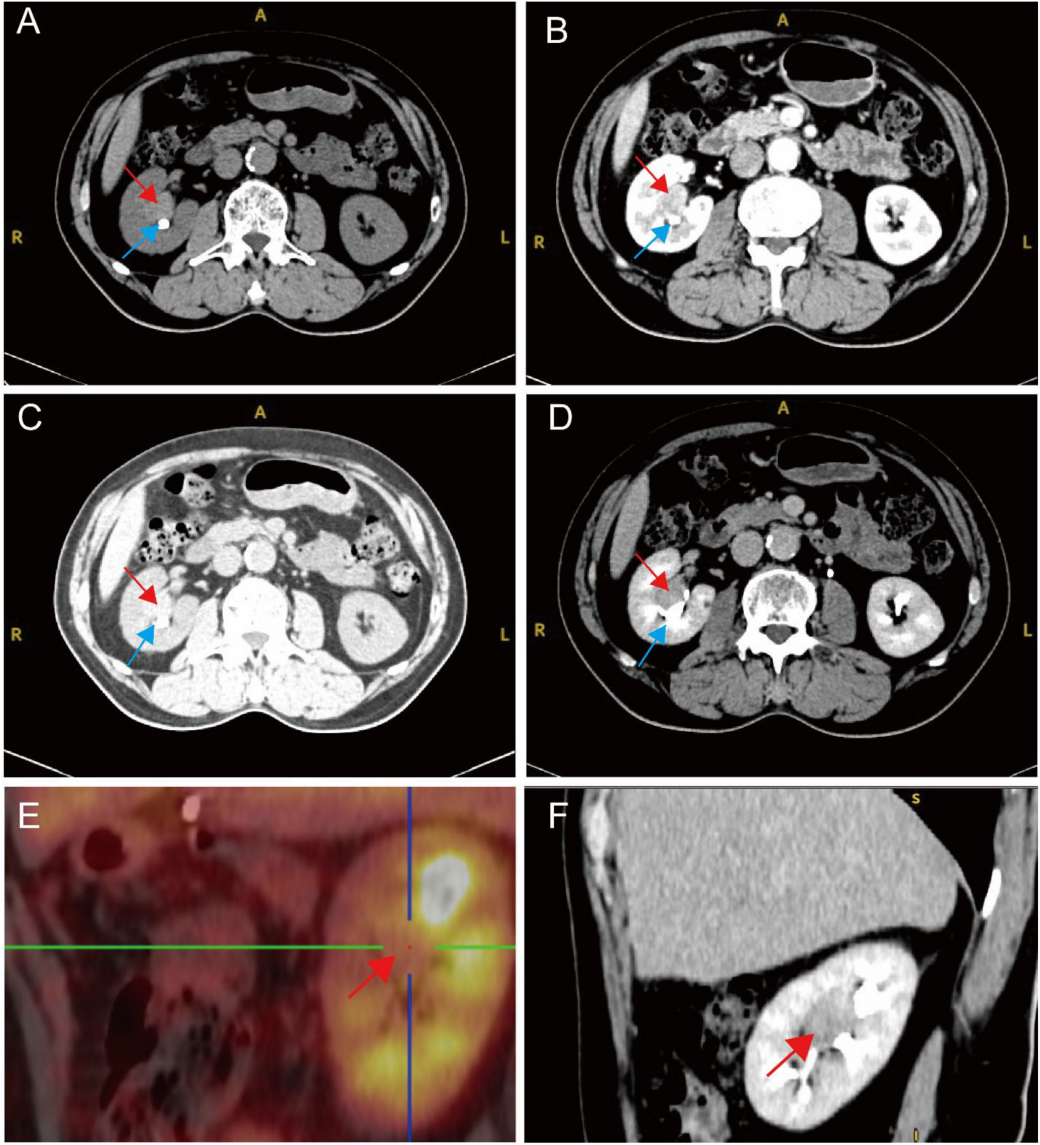
Imaging findings of the right renal mass. **(A)** Non-contrast CT **(axial view)** showing a nodular soft tissue density lesion in the right renal pelvis region measuring 25 mm × 17 mm, with well-defined borders and compression of surrounding adipose tissue (CT value: 51 HU). Red arrow indicates the mass; blue arrowhead indicates the renal pelvis calculus. **(B)** Contrast-enhanced CT in the arterial phase (axial view) demonstrating mild enhancement of the mass (57 HU). Red arrow indicates the mass; blue arrowhead indicates the renal pelvis calculus. **(C)** Contrast-enhanced CT in the corticomedullary phase (axial view) showing marked enhancement of the mass (102 HU). Red arrow indicates the mass; blue arrowhead indicates the renal pelvis calculus. **(D)** Contrast-enhanced CT in the excretory phase (axial view) demonstrating moderate enhancement of the mass (74 HU) with a filling defect in the right renal pelvis. Red arrow indicates the mass; blue arrowhead indicates the renal pelvis calculus. **(E)** PET-CT (sagittal view) revealing increased FDG uptake in the right renal hilar mass with SUVmax of 2.6, indicating increased glucose metabolism. Red arrow indicates the mass. **(F)** Contrast-enhanced CT in the excretory phase (sagittal view) showing opacification of the renal calyces with a filling defect in the renal pelvis region, demonstrating non-visualization of contrast material in the area occupied by the mass. Red arrow indicates the mass.

Renal dynamic imaging showed normal bilateral renal blood perfusion and function, with left kidney GFR 42.78 ml/min, right kidney GFR 39.38 ml/min, and total GFR 80 ml/min. Non-contrast CT (**Figure 3A**) revealed a nodular soft tissue density lesion in the right renal pelvis region with a CT value of approximately 51 HU, measuring approximately 25 mm × 17 mm at its largest cross-section, with well-defined borders and compression of surrounding adipose tissue. Contrast-enhanced CT (**Figures 3B–D**) demonstrated mild enhancement during the arterial phase (57 HU), marked enhancement during the corticomedullary phase (102 HU), and moderate enhancement during the excretory phase (74 HU). A filling defect in the right renal pelvis was clearly visualized on both axial (**Figure 3D**) and sagittal (**Figure 3F**) excretory phase images, with opacification of the renal calyces but non-visualization of the renal pelvis in the mass region. PET-CT (**Figure 3E**) revealed a solid nodule in the right renal hilar region, measuring approximately 21 mm × 18 mm, with SUVmax of 2.6, indicating increased glucose metabolism. Cystoscopy showed clear urine efflux from both ureteral orifices and normal bladder mucosa. CT urography (CTU) was not separately performed, as the excretory phase of the contrast-enhanced CT provided equivalent functional information for evaluating the collecting system. The excretory phase images (**Figures 3D, F**) clearly demonstrated opacification of the renal calyces with a filling defect in the right renal pelvis region where the mass was located, indicating obstruction of contrast material flow by the tumor. Chest X-ray and electrocardiogram showed no abnormalities.

### 2.4 Treatment strategy and prognosis

On November, 2024, a multidisciplinary team (MDT) consultation involving urology, radiology, pathology, nuclear medicine, and anesthesiology was conducted with comprehensive analysis: the patient was an elderly male with high-risk factors for UTUC (long-term smoking, history of renal calculi), presenting with painless gross hematuria as the main manifestation. Imaging studies revealed an occupying lesion in the right renal pelvis region with filling defect during the excretory phase, PET-CT suggested a neoplastic lesion, and urine cytology did not exclude tumor cells. The preliminary diagnosis was UTUC.

Regarding preoperative biopsy: considering the patient’s advanced age, multiple comorbidities, and relatively poor general condition; ureteroscopic biopsy carried risks of tumor dissemination, bleeding, and infection, and might require repeat anesthesia. After thorough weighing of benefits and risks and communication with the patient, the decision was made to forgo ureteroscopic biopsy.

On December, 2024, laparoscopic right radical nephroureterectomy with bladder cuff excision was performed under general anesthesia. The surgery was uneventful, with no perioperative complications.

### 2.5 Pathological examination

Gross examination (**Figure 2A****):** The mass measured 2.6 cm × 2.0 cm × 1.7 cm, soft in consistency, with a tan-brown cut surface, ill-defined margins, originating from the renal parenchyma and involving the renal sinus fat.

Microscopic findings (**Figures 2C, E, G****):** At low magnification (**Figure 2E**, ×10), the tumor originated from the renal parenchyma with expansile growth pattern, extending into the renal sinus and perirenal fat tissue. The tumor compressed but did not invade the renal pelvis, and showed a well-circumscribed border with pushing margins rather than infiltrative growth. No obvious tumor capsule was identified. Tumor cells were arranged in solid nests with abundant intervening vasculature, with some vessels showing dilatation and congestion (**Figure 2G**, ×20). At high magnification (**Figure 2C**, ×40), tumor cells were large with distinct cell borders, abundant eosinophilic granular cytoplasm with focally slightly clear areas, and characteristic intracytoplasmic vacuoles. Nuclei were round to oval with variable sizes, some showing irregular nuclear membranes, unevenly distributed chromatin, and prominent nucleoli. Mitotic figures were rare.

Immunohistochemistry (**Figure 2D, F, H**, ×40): CD117 (+) with cytoplasmic and membranous staining (**Figure 2D**), PAX8 (+) with nuclear staining (**Figure 2H**), CK7 (–) (**Figure 2F**), CAIX (–), RCC (–), CD10 (-), Vimentin (-), TFE3 (-), SDHB (+) (retained expression), P63 (-), Ki-67 proliferation index approximately 1%.

Based on histological morphology and immunophenotype, the pathological diagnosis was: Eosinophilic Vacuolated Tumor (EVT).

### 2.6 Follow-up

At 9-month postoperative follow-up, the patient had recovered well with normal renal function, and imaging studies showed no evidence of tumor recurrence or distant metastasis.

## 3 Discussion

### 3.1 Evolution of understanding and epidemiological features of EVT

The nomenclature of EVT has undergone gradual evolution. In 2018, He et al. (1) first reported 14 cases of renal tumors composed of oncocytic cells with high-grade nuclei and vacuolated cytoplasm, proposing the term “high-grade oncocytic tumor.” In 2019, Chen et al. (2) discovered TSC/mTOR signaling pathway activation mutations in this type of tumor through targeted gene sequencing, describing it as “sporadic renal cell carcinoma with eosinophilic and vacuolated cytoplasm.” In 2021, GUPS unified its nomenclature as eosinophilic vacuolated tumor (3), and the 2022 WHO 5th edition Classification of Urinary and Male Genital System Tumors acknowledged it under the category of other oncocytic renal tumors (4).

To date, approximately 84 cases of EVT have been reported in the literature (including the present case, based on searches of PubMed, Web of Science, and CNKI databases as of September 2025; see **Table 1**). Epidemiological features show: female predominance over male (50:34), age range 14–79 years, median age 50 years, tumor diameter 1.5-11.2 cm. Most patients have no specific clinical manifestations and are often discovered incidentally during physical examination, with a minority presenting with lumbago (9) or hematuria (10). The vast majority are sporadic, with only a few occurring in patients with tuberous sclerosis complex (11, 12).

**Table 1:** Summary of EVT cases reported in the literature.

Year	Author	Sex	Location	Age (years)	Presenting Symptoms	Treatment	Postoperative Follow-up
2025	Coiner et al. (16)	F	1 Right	27	Hematuria with flank pain	Radical nephrectomy (hilar LN 3/5 positive)	NED at 4.5 months
2025	Hu et al. (6)	M2:F2	2 Left:2 Right	35-54	Incidental finding	Partial nephrectomy	NED at 2–11 months
2025	Liang et al. (18)	M1:F2	3 Right	32-70 (mean 47)	Incidental finding	Partial nephrectomy	NED at 2–12 months
2025	Ou et al. (8)	F1	1 Left	52	Incidental finding	Partial nephrectomy	NED at 48 months
2025	Niu et al. (19)	F1	1 Right	67	Incidental finding	Laparoscopic tumor enucleation + perirenal adhesiolysis + perirenal lymphadenectomy	NED at 14 months
2025	Fu et al. (20)	M1	1 Right	52	Incidental finding	Partial nephrectomy	NED at 11 months
2025	Liu et al. (7)	M1	1 Right	14	Incidental finding	Radical nephrectomy	Mediastinal LN metastasis at 54 months
2024	Choinieren et al. (21)	M3:F2	2 Right:3 Left	46-63 (mean 55)	Not specified	Active surveillance (2/5) or nephrectomy (3/5)	NED at 8–90 months
2024	Wang et al. (14)	M4:F3	Not specified	29-61 (mean 42)	Incidental finding	6 partial nephrectomy, 1 radical nephrectomy	NED at 4–96 months
2023	Zhang (22)	F1	1 Left	56	Incidental finding	Partial nephrectomy	NA
2023	Chen et al. (23)	F1	1 Left	33	Lumbar discomfort	Radical nephrectomy	NED at 12 months
2023	Liu et al. (10)	F1	1 Right	60	Hematuria, dysuria	Partial nephrectomy	NED at 23 months
2023	Chen et al. (24)	F1	1 Right	60	Incidental finding	Partial nephrectomy	NED at 12 months
2023	Dong et al. (25)	F2	1 Right:1 Left	60, 79	Incidental finding	1 partial, 1 radical nephrectomy	NED at 6–8 months
2022	Kartal et al. (9)	M1	1 Left	47	Lumbar discomfort	Partial nephrectomy	NED at 6 months
2022	Amin et al. (11)	M1	Left: unclassified RCC; Right: EVT	44	TSC patient, bilateral renal masses	Surgical resection (not specified)	NA
2022	Zhang et al. (5)	M2:F2	4 Left	44-63 (mean 51)	3 incidental, 1 lumbar discomfort	1 radical, 3 partial nephrectomy	NED at 10–88 months
2022	Farcaş et al. (15)	M8:F11	Not specified	15-72 (mean 47, median 50)	Incidental finding	Surgical resection (not specified)	NED at 12–198 months
2022	Hu et al. (26)	M2:F3	Not specified	25-62 (mean 44.4, median 45)	Not specified	Partial/radical nephrectomy (not specified)	NED at 11–86 months
2021	Kapur et al. (27)	M1	1 Left	55	Incidental finding	Partial nephrectomy	NED at 15 months
2019	Chen et al. (2)	M3:F4	2 Left:5 Right	40-68 (mean 54)	Incidental finding	Partial nephrectomy	NED at 14 months
2019	Trpkov et al. (12)	F1	1 Right, Left pending	48	TSC patient, bilateral renal masses	Radical nephrectomy	NA

M, male; F, female; LN, lymph node; NED, no evidence of disease; NA, not available; TSC, tuberous sclerosis complex; RCC, renal cell carcinoma; EVT, eosinophilic vacuolated tumor.

### 3.2 Imaging features and differential diagnosis

EVT lacks typical imaging features. In previous reports, EVT mostly presents as a mass in the parenchymal region of the upper or lower pole of the kidney (5, 6). Ou et al. (8) reported a case where CT suggested mild tumor invasion of the renal sinus causing mild sinus deformity, but presentation as a mass involving the renal pelvis region mimicking UTUC has not been previously reported.

The imaging features of this case are distinctive: non-contrast CT showed a nodular soft tissue density lesion in the renal pelvis region (51 HU) with well-defined borders and an endophytic growth pattern confined to the kidney; contrast-enhanced CT demonstrated an enhancement pattern of “mild arterial phase - marked corticomedullary phase - moderate excretory phase” (57/102/74 HU), with a filling defect of the renal pelvis visible during the excretory phase. This appearance is highly similar to the typical features of UTUC, leading to preoperative misdiagnosis. However, careful analysis reveals important inconsistencies: the lesion had well-defined, smooth borders rather than the irregular, infiltrative margins typical of UTUC, and showed marked corticomedullary phase enhancement (102 HU), suggesting rich blood supply more consistent with a tumor of renal parenchymal origin rather than the relatively hypovascular urothelial tumor. The well-defined margins and endophytic growth pattern observed in this case are characteristic features of renal parenchymal neoplasms and should have raised suspicion for an alternative diagnosis despite the presence of a filling defect. Additionally, PET-CT in this case showed SUVmax of 2.6, representing mild glucose metabolic activity. Literature reports that the mean SUVmax of primary UTUC is 15.4 ± 13.8 (range 2.8-43.4) (13), and the SUVmax in this case was significantly lower than the average for UTUC. Although definitive conclusions cannot be drawn from a single case, the relatively lower metabolic activity may provide some reference clues for preoperative differential diagnosis.

Imaging differential diagnosis should consider the following tumors: ① Clear cell renal cell carcinoma: mostly originates from renal cortex, often shows exophytic growth with irregular margins, with obvious internal heterogeneity, commonly with hemorrhage, necrosis, cystic change, or calcification, and demonstrates a “fast in, fast out” hypervascular enhancement pattern. ② Renal oncocytoma: shows expansile exophytic growth with well-defined borders, may have a pseudocapsule, relatively homogeneous enhancement, and some may show delayed enhancement of a central scar. ③ UTUC: lesion epicenter located within the renal pelvis cavity or arising from the urothelial lining, typically presenting as an irregular or papillary mass causing a filling defect within the renal pelvis cavity, with mild to moderate enhancement intensity generally lower than renal parenchyma during the corticomedullary phase, and characteristically demonstrates irregular or infiltrative margins rather than smooth, well-defined borders.

### 3.3 Pathological features and differential diagnosis

The pathological features of EVT are relatively distinctive. Grossly, the tumor presents as a well-circumscribed solid nodule with pushing margins but without a true capsule, with a tan-brown cut surface and texture similar to normal renal parenchyma (1, 14). Microscopic features include: tumor cells arranged in solid nests, some with tubular-cystic architecture; large cells with abundant, eosinophilic, granular cytoplasm and characteristic vacuoles; nuclei with high-grade morphology and prominent nucleoli; stroma may show hyalinization, calcification, or loose edema (1).

Regarding immunophenotype, CD117 positivity and CK7 negativity are classic features of EVT. Additionally, PAX8, Cathepsin K, CD10, and SDHB are usually positive, while CAIX, TFE3, Vimentin, and HMB-45 are mostly negative. Molecular genetics commonly show TSC/mTOR pathway mutations (2, 15). In this case, immunohistochemistry showed CD117 (+), CK7 (–), PAX8 (+), SDHB (+), TFE3 (–), Ki-67 approximately 1%, consistent with EVT diagnostic criteria. Cathepsin K staining was not performed in this case. Notably, CD10 was negative in this case. In previous studies, He et al. (1) reported that 2 of 14 high-grade oncocytic tumors were CD10 negative, with 1 case also being Cathepsin K negative, suggesting some immunophenotypic heterogeneity in EVT. The diagnosis of EVT can be established based on the constellation of morphological features and the characteristic immunophenotypic profile (CD117+/CK7-/PAX8+) without requiring Cathepsin K staining.

Pathological differential diagnosis should note: ① Oncocytoma: may have fibrous scar, round and regular nuclei, inconspicuous nucleoli, immunophenotype CD117 (+)/CK7 (–), main differences being that EVT has prominent nucleoli, more significant cellular pleomorphism, and characteristic cytoplasmic vacuoles. ② Chromophobe renal cell carcinoma: eosinophilic cytoplasm with perinuclear halo, wrinkled and folded nuclei, CK7 positive. ③ MiT family translocation-associated renal cell carcinoma: TFE3/TFEB, HMB-45, MelanA positive. ④ SDH-deficient renal carcinoma: SDHB expression loss. ⑤ FH-deficient renal cell carcinoma: FH negative, 2SC positive.

### 3.4 Treatment strategy and prognosis

Currently, the standard treatment protocol for EVT has not been established. Given its favorable biological behavior, nephron-sparing surgery (partial nephrectomy) should be the first choice for well-circumscribed tumors confined to the kidney with no evidence of metastasis. Previous large series reports show that EVT has a good prognosis, with recurrence or metastasis being rare (1, 14, 15). However, Liu et al. (7) reported the first case of EVT with mediastinal lymph node metastasis 54 months after surgery, and the case reported by Coiner et al. (16) showed aggressive histological features including lymphovascular invasion, perineural invasion, and renal sinus fat invasion. These findings suggest that EVT may have certain malignant potential, and long-term follow-up remains necessary.

Although the tumor in this case involved the renal sinus fat, no recurrence was observed at 9-month postoperative follow-up. Considering that a definitive pathological diagnosis was not obtained preoperatively, radical nephroureterectomy was performed based on the diagnosis of UTUC, which extended the surgical scope. This lesson suggests: for renal pelvis masses with atypical imaging features, especially those with marked corticomedullary phase enhancement suggesting a tumor of renal parenchymal origin, preoperative biopsy should be actively considered. Percutaneous biopsy is an effective means of determining the pathological type of renal parenchymal tumors; for cases highly suspicious for UTUC, ureteroscopic biopsy followed by chemotherapy to reduce tumor dissemination risk may be considered (17).

## 4 Conclusion

This article reports a rare case of EVT misdiagnosed as UTUC. The distinctive feature of this case is that although the tumor originated from the renal parenchyma, involvement of the renal sinus resulted in imaging presentation as a renal pelvis mass, making it difficult to differentiate from UTUC. Our lessons learned indicate that: when a renal pelvis mass shows marked corticomedullary phase enhancement, the possibility of a tumor of renal parenchymal origin should be considered; preoperative biopsy is of great value for establishing a definitive pathological diagnosis and formulating an appropriate surgical plan. Given the limited number of reported EVT cases and insufficient long-term prognostic data, future multicenter collaboration to accumulate cases is still needed to further elucidate its biological behavior and refine.

## Data Availability

All original data involved in this study are included in the article. For further inquiries, please contact the corresponding author.
